# The Relationship Between COVID‐19 Vaccine Regret, Distrust of the Healthcare System, and Future Vaccine Intentions

**DOI:** 10.1111/jep.70532

**Published:** 2026-07-19

**Authors:** Ali Tayhan, Bilge Azakli

**Affiliations:** ^1^ Department of Nursing, Faculty of Health Sciences Manisa Celal Bayar University Manisa Turkey

**Keywords:** COVID‐19 vaccine regret, health system distrust, intention to get vaccinated

## Abstract

**Background:**

Vaccines are one of the most effective interventions used by public health services to reduce infectious diseases. However, individuals' attitudes toward vaccines are also crucial to vaccine effectiveness. Experiences with vaccines and trust in healthcare services can guide future vaccination decisions.

**Aims:**

The aim of this study was to examine the relationship between COVID‐19 vaccine regret, distrust of the healthcare system, and future vaccination intentions.

**Methods:**

In this cross‐sectional study with a qualitative component, data were obtained through face‐to‐face interviews (*n* = 402). Data were collected using the Decision Regret Scale (DRS), the Health System Distrust Scale (HCSDS), and open‐ended questions regarding the reasons for COVID‐19 vaccine regret. To ensure clarity of the findings, quantitative and qualitative data were integrated.

**Results:**

Of the participants in the study, 87.1% did not experience any adverse effects from the COVID‐19 vaccine. 56.2% of participants regretted getting the COVID‐19 vaccine. Additionally, 35.8% reported being ‘undecided’ about accepting a new vaccine in the future. Among participants, it was found that experiencing a side effect after the COVID‐19 vaccine increased distrust in the healthcare system (*p* < 0.05). A weak positive correlation was found between participants' HCSDS and DRS scores (*r* = 0.282, *p* < 0.001). Qualitative analyses showed that participants' regrets regarding the COVID‐19 vaccine were mainly grouped around two themes: (1) ‘Concern about long‐term effects’, (2) ‘Perception that the vaccine is unnecessary’.

**Conclusions:**

Based on these findings, it can be said that the phenomenon of COVID‐19 vaccine regret and the level of distrust in the healthcare system will make it even more difficult to control future pandemics and infectious diseases. In addition, the findings highlight the need for communication strategies aimed at increasing trust in vaccines and the healthcare system.

## 1 Introduction

Vaccines are beneficial public health interventions to prevent death and disability from infectious diseases and to improve quality of life. Vaccination programmes are implemented for children and adults in many countries around the world [1]. Indeed, the effectiveness of vaccines has been proven during the COVID‐19 pandemic. However, there has been an increase in vaccine hesitancy in recent years. Vaccine hesitancy is a growing obstacle to vaccination programmes and has a complex structure [2, 3]. Factors such as conspiracy theories, perceptions of distrust, and negative experiences have the potential to influence individuals' vaccination decisions. Furthermore, it is known that the COVID‐19 pandemic has affected public attitudes towards vaccines [4]. Leonardelli et al. report that vaccine hesitancy has gained a new dimension with the COVID‐19 pandemic and emphasise that new variables affecting vaccine hesitancy have begun to emerge after the pandemic [5]. Ortiz‐Prado et al. (2025) report that although COVID‐19 vaccines have saved millions of lives, they have become a paradox that forms the basis of the increasingly powerful anti‐vaccine movement today. They argue that this paradox created by the COVID‐19 vaccination campaign needs to be thoroughly investigated in terms of its future effects [6]. Skirrow et al. (2024) report that some individuals are more hesitant about routine vaccination after the pandemic [7]. Similarly, Evens et al. report that vaccine hesitancy tends to increase after the pandemic [8]. Matos et al. (2025) found that parents' trust in childhood vaccinations decreased after the pandemic [9]. In addition, a recent study on vaccine hesitancy highlights the phenomenon of COVID‐19 vaccine regret and its potential future effects [10]. Brera et al. define the concept of COVID‐19 vaccine regret as a feeling of distress and regret that an individual feels at any time for any reason after receiving the COVID‐19 vaccine. This is the thought, ‘I wish I hadn't gotten the vaccine.’ Regret is a powerful emotion that can influence people's future decisions and attitudes. This feeling creates a tendency to avoid past decisions and remains in memory for a long time. In this context, COVID‐19 vaccine regret can have consequences for future vaccination decisions and behaviours and can become a leverage for vaccine hesitancy [11, 12].

Another factor related to attitudes toward vaccines is the perception of distrust. Research shows that major crises can affect public trust in authorities and existing systems. Given that the COVID‐19 pandemic was a major health crisis, it can be expected that public trust in the health system will change positively or negatively, and this will have some consequences in the future [13]. How healthcare services fare in this test in the eyes of the public is important. The perception of distrust affects the acceptance of healthcare services. In particular, primary healthcare services and vaccination programmes rely on the perception of trust [14].

Debbag et al. (2025) state that mandatory vaccination and various restrictions implemented during the COVID‐19 pandemic affected public trust in authorities and the health system, and such situations may affect trust in health services and acceptance of vaccination programmes in future potential pandemics [15].

The aim of this study is to examine the relationship between individuals' regret about getting the COVID‐19 vaccine, their trust/distrust in the healthcare system, and their intention to get vaccinated in the future.

In the research, we meticulously sought answers to the following questions:

- Is there a relationship between COVID‐19 vaccine regret and individuals' future vaccination intentions?
- Is there a relationship between the level of distrust in the healthcare system after the pandemic and individuals' intentions to get vaccinated in the future?
- Is there a relationship between COVID‐19 vaccine regret and distrust in the healthcare system?

## 2 Methods

This cross‐sectional study with a qualitative component was conducted between January 10, 2024, and October 15, 2024, within the service area of a public health centre located in western Türkiye. According to the population registration system, there were 136,000 people in the research area. According to these population data, the sample size was calculated as 383 with 5% error and 95% confidence interval. Considering potential data loss, a 5% increase was applied to the calculated sample size, resulting in a final sample of 402 participants.

For the data collection process, street numbers of the research area were obtained from the local municipality (There were a total of 1603 non‐repeating street numbers). All street numbers were coded in Microsoft Excel. In the column next to each street number, a random number was generated using the Excel programme's ‘random number generation’ formula. The random numbers were not repeating. These randomly generated numbers were matched with street numbers. After the matching, the random numbers were sorted from smallest to largest. Next, the street numbers that matched the first 402 random numbers in the sequence were identified. These procedures were carried out to increase the randomness of the data collection process.

During the data collection process, in these identified streets, the first house on the right side at the beginning of the street was visited. If there was no one in the visited household or no one volunteered to participate in the study, the next household on the same street was visited. Only one volunteer from each house was included in the study. Inclusion criteria for the study were: being over 18 years of age, being able to read and write Turkish, being cognitively healthy, having been vaccinated against COVID‐19, and voluntarily participating in the study. In line with the study's objective, individuals who received the COVID‐19 vaccine were included in the research to assess their regret about the vaccine. Therefore, those who did not receive the COVID‐19 vaccine were not included in the study. Data was collected face to face by the researchers using the following data collection forms. Data collection time lasted between 8 and 10 min.

### 2.1 Data Collection Instruments

The Sociodemographic Information Form consisted of questions developed by the researchers in line with the literature. The form included questions such as age, gender, COVID‐19 vaccine preference, vaccine side effects, COVID‐19 disease history, intention to get vaccinated in the future (for a new pandemic), etc. In addition, open‐ended questions were included in the data collection form to determine the reasons for regretting the vaccination decision. Although this study has a cross‐sectional design, it was planned to enrich the research data with these open‐ended questions. The open‐ended questions were: Do you regret getting the COVID‐19 vaccine? If yes, please explain the reasons for your regret regarding the COVID‐19 vaccine.

Decision Regret Scale (DRS); DRS, developed by Brehaut et al. (2003), determines the level of regret felt for a decision taken [16]. Telatar et al. (2021) adapted DRS to Turkish [17]. The scale was used in this study to determine individuals' COVID−19 vaccination decision regret levels. The scale is composed of 5 items. A score of 0 on the scale means that no regret is felt for the decision taken, while a score of 100 indicates the highest level of regret. In the current study, the Cronbach's alpha value of the scale was found to be 0.82.

Health Care System Distrust Scale (HCSDS); The scale developed by Rose et al. (2004) was adapted to Turkish by Yeşildal et al. (2020) [18, 19]. The scale consists of 10 items and a single dimension. Scale score is calculated by the arithmetic mean method. As the scores obtained from the scale approach 5, it indicates that distrust in health systems increases, and as they approach 1, it indicates that trust increases. In the current study, the Cronbach's alpha value of the scale was found to be 0.84.

### 2.2 Analysis

Data was analysed using SPSS 25. Frequencies and percentages of sociodemographic data, mean and standard deviations of scale scores were calculated. The distribution of the data was determined by examining the skewness and kurtosis values. The skewness and kurtosis coefficients of the scale scores were found to be between +/−1, and it was determined that the data were normally distributed [20]. The relationship between sociodemographic data and scale scores was examined with One Way ANOVA test and Student's *t*‐test. The relationship between scale scores was examined using Pearson analysis.

Responses to the open‐ended questions on the data collection form provided valuable insights into understanding the phenomenon of COVID‐19 vaccine regret. Qualitative data were analysed according to the recommendations of Braun and Clarke (2021) [21]. Qualitative analyses were performed using an inductive approach in the NVIVO 12 programme. During the analysis preparation process, the responses written by the participants were transcribed word for word on a computer. These responses were read repeatedly by the researchers to increase their familiarity with the data. Following this process, the analysis was performed. To ensure the reliability of the qualitative analysis, two researchers independently analysed the data and compared the results. Finally, the two researchers reached a consensus on the themes.

To make the findings more meaningful, quantitative and qualitative data were integrated. Data integration was carried out using quantitative data from those who responded to the open‐ended question. Thus, a data integration results matrix was created. The recommendations of Lorenzini et al. (2024) were meticulously applied in integrating the findings [22].

### 2.3 Ethical Consideration

Ethical approval was received from the Health Sciences Ethics Committee of Manisa Celal Bayar University to conduct this research (numbered 2187 and date 27/12/2023). Potential participants were informed about the study, and then verbal and written consent was obtained from those who volunteered. Participants were told that they could withdraw from the study at any time. Detailed information was provided through a voluntary consent form. The principles of the Declaration of Helsinki, ethical rules and human rights were meticulously adhered to at every stage of the research.

## 3 Results

Among the participants in the study, 58.7% were 38 years of age or younger, 52.0% were male, 45.3% were single, 42.0% had income less than expenses, 44.8% were high school graduates, and 27.4% were Freelance (Table 1).

**Table 1:** Sociodemographic variables.

Variables		%	*n* (402)
Age (mean ± SD) 39.12 ± 12.28	38 years and under	58.7	236
	39 years and over	41.3	166
Sex	Female	48.0	193
	Male	52.0	209
Marital status	Married	45.0	181
	Single	45.3	182
	Widow	9.7	39
Income level	Income exceeds expenses	21.4	86
	Income equals expenses	36.6	147
	Income less than expenses	42.0	169
Education level	Elementary school	19.2	77
	High school	44.8	180
	University	36.1	145
Occupation	Unemployed	20.4	82
	Employee	17.7	71
	Officer	18.4	74
	Freelance	27.4	110
	Retired	9.5	38
	Housewife	6.6	27

The information regarding the participants' COVID‐19 disease history and vaccine preferences is as follows; 61.9% of the participants reported that they had not contracted COVID‐19 disease, 54.7% reported that there was someone among their close relatives who had recovered from COVID‐19 disease, 81.4% reported that there was no person they knew who had died of COVID‐19. 62.2% of the participants stated that they preferred the BioNTech brand vaccine, 87.1% reported that they had not experienced any side effects from the COVID‐19 vaccine, 56.2% stated that they regretted getting the COVID‐19 vaccine. 35.8% of participants reported that they were ‘undecided/hesitant’ about accepting a new vaccine in the future (Table 2).

**Table 2:** COVID‐19 disease history and vaccine preference.

Variables		%	*n* (402)
COVID‐19 disease status	Yes, I overcame COVID‐19 disease	38.1	153
	No, I've never had COVID‐19	61.9	249
Has a close relative recovered from COVID‐19?	Yes	54.7	220
	No	45.3	182
Have you ever known someone close to you who died from COVID‐19?	Yes	18.4	74
	No	81.6	328
COVID‐19 vaccine choice	Sinovac	19.4	78
	BioNTech	62.2	250
	Both vaccines	18.4	74
Did the vaccine (COVID‐19) have any adverse effects?	Yes	12.9	52
	No	87.1	350
Do you feel regret about getting vaccinated against COVID‐19?	Yes, I feel regret	56.2	226
	No, I don't feel regret	43.8	176
If a vaccine becomes necessary in the future, what are your intentions to get vaccinated? (for a new pandemic)	Yes, I will get vaccinated (a)	33.8	136
	I'm undecided (b)	35.8	144
	No, I will not get vaccinated (c)	30.3	122

The sociodemographic variables of the participants were compared with the HCSDS and DRS scores. Participants with a history of COVID‐19 disease had statistically significantly lower HCSDS scores. Participants who lost a close relative due to COVID‐19 had significantly lower DRS scores. Participants who received both Sinovac and BioNTech vaccines had significantly lower DRS scores. Participants who experienced COVID‐19 vaccine adverse effects had significantly higher HCSDS and DRS scores than those who did not experience adverse effects. When the participants' future intentions to get vaccinated were compared with the scale scores, the HCSDS and DRS scale scores of those who said ‘yes I will get vaccinated’ were found to be significantly higher than those who said ‘I'm undecided’, and those who said ‘I'm undecided’ were found to be significantly higher than those who said ‘no I will not get vaccinated’ (*p* < 0.05). In the analyses, no significant relationship was found between other variables and scale scores (Table 3).

**Table 3:** Sociodemographic variables and scale scores.

Variables		*n*	HCSDS X̄/SD	DRS X̄/SD
Age	38 years and under	236	3.00 ± 0.85	47.94 ± 36.99
	39 years and over	166	3.02 ± 0.98	51.26 ± 37.70
t/p			0.744/0.45	0.877/0.381
Sex	Female	193	2.97 ± 0.85	51.55 ± 36.82
	Male	209	3.07 ± 0.96	47.24 ± 37.81
t/p			1.123/0.26	1.115/0.24
Marital status	Married	181	3.06 ± 0.95	50.71 ± 37.67
	Single	182	2.98 ± 0.87	48.70 ± 37.45
	Widow	39	3.07 ± 0.89	45.64 ± 36.07
F/p			0.327/0.67	0.339/0.71
Income level	Income exceeds expenses	86	2.82 ± 1.01	42.73 ± 38.39
	Income equals expenses	147	3.11 ± 0.99	54.31 ± 38.09
	Income less than expenses	169	3.05 ± 0.76	48.31 ± 35.77
F/p			2.877/0.06	2.73/0.07
Education level	Elementary school	77	3.17 ± 0.95	55.45 ± 38.05
	High school	180	3.00 ± 0.94	49.80 ± 38.00
	University	145	2.98 ± 0.84	45.44 ± 35.94
F/p			1.075/0.27	1.839/0.16
Occupation	Unemployed	82	3.00 ± 0.82	45.85 ± 34.31
	Employee	71	3.05 ± 1.01	56.19 ± 39.68
	Officer	74	2.80 ± 0.87	47.56 ± 38.10
	Self‐employment	110	3.16 ± 0.93	51.50 ± 36.23
	Retired	38	2.94 ± 0.80	42.10 ± 37.57
	Housewife	27	3.20 ± 1.03	47.77 ± 41.93
F/p			1.344/0.15	1.023/0.40
COVID‐19 disease status	Yes, I overcame COVID‐19 disease	153	2.89 ± 0.83	50.78 ± 35.99
	No, I've never had COVID‐19.	249	3.11 ± 0.95	48.41 ± 38.22
t/p			2.36/0.01	0.61/0.62
Has a close relative recovered from COVID‐19?	Yes	220	3.03 ± 0.92	49.06 ± 38.12
	No	182	3.01 ± 0.89	49.60 ± 36.51
t/p			0.192/0.84	0.146/0.88
Do you have a close relative who died due to COVID‐19?	Yes	74	2.81 ± 0.88	45.94 ± 38.73
	No	328	3.02 ± 0.81	50.07 ± 37.06
t/p			0.136/0.60	0.859/0.02
COVID‐19 vaccine choice	Sinovac (a)	78	2.97 ± 0.83	51.15 ± 34.08
	BioNTech (b)	250	3.05 ± 0.94	50.28 ± 38.20
	Both vaccines (c)	74	2.98 ± 0.89	44.12 ± 37.82
F/p			0.356/0.70	0.89/0.04
Post‐hoc*				c < a = b
Did the vaccine (COVID‐19) have any adverse effects?	Yes	52	3.42 ± 0.82	72.69 ± 29.91
	No	350	2.97 ± 0.91	45.84 ± 37.17
t/p			3.397/0.00	4.94/0.00
If a vaccine becomes necessary in the future, what are your intentions to get vaccinated? (for a new pandemic)	Yes, I will get vaccinated (a)	136	2.50 ± 0.69	24.59 ± 25.73
	I'm undecided (b)	144	3.13 ± 0.83	52.11 ± 35.00
	No, I will not get vaccinated (c)	122	3.49 ± 0.92	73.56 ± 33.91
F/p			44.88/0.00	71.26/0.00
Post‐hoc*			a < b < c	a < b < c

*Note: t*, Student's *t*‐test; *F*, One Way ANOVA test; *, Tukey HSD.

A weak positive significant relationship was found between the participants' levels of decision regret regarding the COVID‐19 vaccine and their levels of distrust in the healthcare system (*r* = 0.282, *p* < 0.001) (Table 4).

**Table 4:** Relationship between HCSDS and DRS scores (*n* = 402).

Variables	Pearson	HCSDS	DRS
HCSDS	Correlation coefficient	1	0.282
	p	—	< 0.000
DRS	Correlation coefficient	0.282	1
	p	< 0.000	—

### 3.1 Qualitative Findings Regarding Open‐Ended Questions

In the study, participants who experienced COVID‐19 vaccine regret were asked about the reasons for this. A total of 214 participants wrote answers to these questions. Two themes emerged from the qualitative analysis of participants' statements.

#### 3.1.1 Theme 1: Concern About Long‐Term Effects

The majority of participants who expressed regret were concerned about the future effects of the vaccine (*f* = *132, 61,6%*). They reported that this uncertainty led to feelings of regret. It could be said that underlying this theme are concerns, threats, and fears about the future. This theme includes the belief that vaccines have various hidden agendas for human health. Some of the participant responses reflected conspiracy theories regarding the COVID‐19 vaccine.

##### 3.1.1.1 Some Quotes

I don't know what consequences the vaccine will have in the future. Over the years, I've become concerned about how this vaccine will affect our bodies.

(Survey Number: 185, Male)

From what I learned from social media, the vaccine may cause different health problems in the future… This worries me.

(Survey Number: 394, Male)

In discussion programmes, it was mentioned that the vaccine was produced in a very short time. Its effects and consequences may not have been well researched.

(Survey Number: 115, Female)

#### 3.1.2 Theme 2: Perception That the Vaccine Is Unnecessary

Participants stated that they contracted COVID‐19 despite receiving the COVID‐19 vaccine and that the vaccine did not provide sufficient protection (*f* = *82, 38,3%*). This theme highlights the disappointment surrounding the vaccine's effectiveness. The perception that expectations for the vaccine have not been met is particularly noteworthy.

##### 3.1.2.1 Some Quotes

I got sick even though I was vaccinated. It didn't provide any protection.

(Survey Number: 207, Male)

From what I've seen on the news, some people who were vaccinated have also died from the disease. This vaccine was a disappointment. It didn't seem to offer enough protection.

(Survey Number: 75, Female)

I got sick even though I was vaccinated. My mother wasn't vaccinated, but she didn't get sick. I didn't see any difference between those who were vaccinated and those who weren't.

(Survey Number: 154, Female)

### 3.2 Integration of Quantitative and Qualitative Findings

When quantitative and qualitative findings were integrated, it was observed that participants who identified with the theme of ‘**Concern about long‐term effects**’ had high scores for vaccine regret and distrust of the health system. Participants who identified with the theme of ‘**Perception that the vaccine is unnecessary**’ had relatively lower scores on the health system trust scale (Table 5).

**Table 5:** Joint display (Integrated results matrix).

DRS (X̄)	HCSDS (X̄)	Theme	Interpretation	
3,26	56,31	**Theme 1:** *Concern about long‐term effects (f* = *132, 61,6%)*	Concerns about the long‐term effects of Covid‐19 vaccines are emerging as a significant reason for both vaccine regret and distrust in the healthcare system.	*It can be said that both themes provide similar levels of justification for vaccine regret. However, when the relationship between the issues of distrust in the healthcare system and these themes is examined, it is clear that there is a divergence between the two themes. It can be argued that anxiety and fear about the future effects of the vaccine (Theme 1) leads to greater distrust in the healthcare system than disappointment with vaccines (Theme 2)*.
3,21	50,03	**Theme 2:** *Perception that the vaccine is unnecessary (f* = *82, 38,3%)*	According to quantitative data, the second theme leads to relatively less vaccine hesitancy and distrust of the healthcare system compared to the first theme.	

## 4 Discussion

Vaccines are among the effective interventions used by healthcare services in combating various infectious diseases. The effectiveness of vaccines has also been demonstrated during the COVID‐19 pandemic. However, some studies suggest that individuals' trust in vaccines and the health system has been affected in various ways by the pandemic [7, 23]. In this context, it can be said that identifying trends in society regarding vaccination and the healthcare system is important in terms of infectious diseases and potential new pandemics. This study examined the relationship between individuals' regret about the COVID‐19 vaccine, their levels of trust/distrust in the healthcare system, and their future intentions to get vaccinated.

To better interpret the findings, it would be helpful to mention the context in which the research was conducted. During the pandemic, COVID‐19 vaccination was not legally mandatory in Türkiye. However, it was mandatory to have a vaccination card or a negative PCR test result obtained within the last 7 days to enter all government buildings, shopping malls, and use public transportation. These practices can be said to be an indirect and powerful incentive for COVID‐19 vaccines [24]. During the pandemic, licensed COVID‐19 vaccines were systematically distributed throughout Türkiye by the Ministry of Health. Sinovac was the first vaccine to become available, followed by the BioNTech vaccine approximately 3–6 months later. Both vaccines were administered free of charge in hospitals and family health centres. By the end of the pandemic, the coverage of at least one dose of a COVID‐19 vaccine had reached 93% in Türkiye [24, 25].

The study found that more than half of the participants experienced varying degrees of regret for having received the COVID‐19 vaccine. Partially similarly, a study conducted by Hussein et al. among healthcare workers in Iraq reported over 30% regret about the COVID‐19 vaccine [26]. In fact, given that public attitudes towards vaccines in Türkiye were generally positive before the pandemic, and that COVID‐19 vaccines saved many lives, this high level of regret regarding the COVID‐19 vaccine is surprising [27]. At this point, when looking at the qualitative findings, the emerging themes reveal important data about the reasons for vaccine regret. One of these is the theme of ‘Concern about long‐term effects.’ This theme is based on feelings of uncertainty, risk perception, and anxiety regarding vaccine regret. The potential future adverse effects of the vaccine appear to be a significant influencer of vaccine regret. Similarly, Salazar‐Fernández et al. (2023) report that individuals have concerns and fears about the unknown side effects of the COVID‐19 vaccine and about the development stages of the vaccine [28]. In a study conducted in Germany, Fiske et al. (2022) found that the relatively rapid development of the COVID‐19 vaccine raised concerns about the safety and future outcomes of the vaccine [29]. In light of this data, it can be said that uncertainties about the long‐term effects of the vaccine fuel feelings of regret. Conducting research that reveals the long‐term effects of the vaccine and presenting these research findings transparently to the public can reduce feelings of regret towards the COVID‐19 vaccine. Another important theme is ‘Perception that the vaccine is unnecessary’. This theme is based on unmet expectations and disappointment. It can be said that doubts about the protective effect of the COVID‐19 vaccine are another important factor triggering COVID‐19 vaccine regret [30]. To reduce this situation and gain confidence, the results of retrospective research on the protective effect of COVID‐19 vaccines during the pandemic can be shared with the public. In this way, it can be shown to the public how many lives the vaccines have saved. The findings suggest a need to improve public‐targeted communication strategies within healthcare services.

When participants' COVID‐19 vaccine regret scale scores and sociodemographic variables were examined, it was observed that participants who lost a loved one due to COVID‐19 had lower levels of COVID‐19 vaccine regret. This may be related to risk perception. Those who had lost a loved one may have considered the vaccine to be an effective method of protection against the severity of the disease. This may have strengthened their belief that their decision to get vaccinated was the right one. It can be said that the fatal outcome of the disease supports confidence in vaccines [26].

When vaccine preferences and regret levels were examined, those who received both Sinovac and BioNTech brand vaccines had significantly lower levels of vaccine regret. This may be related to the perception of enhanced protection. The fact that individuals accepted both vaccines recommended by health authorities to protect themselves from the disease may indicate that they did not have concerns about vaccine safety and future effects, and that they strongly decided to get vaccinated [31]. It can be said that their levels of regret are low as a natural consequence of such a decision‐making process.

In the study, those who experienced adverse effects from the COVID‐19 vaccine had a higher level of vaccine regret than those who did not experience adverse effects. Similarly, Luo et al. (2022) found that people who experienced vaccine adverse effects felt more regret about taking the vaccine than those who did not experience adverse effects [32]. It is known that adverse effects that occur during treatment are the most common cause of regret [33]. Similar findings are reported in the literature for many preventive practices. Adverse effects of protective and therapeutic practices can often cause feelings of regret in individuals. In general, informing individuals about the possible adverse effects of vaccines and treatments can alleviate their feelings of regret [34].

In the study, when comparing the intention to get vaccinated in the event of a possible future pandemic with the level of regret about the COVID‐19 vaccine, participants who answered ‘No, I will not get vaccinated’ had higher levels of vaccine regret. These data demonstrate the potential of vaccine regret to influence future vaccination decisions. Similarly, Brera et al. (2023) state that vaccine regret may play a significant role in shaping attitudes toward new vaccines in the future [11]. Luo et al. found that healthcare workers who regretted receiving the COVID‐19 vaccine were less likely to accept booster doses [32]. The findings of these three studies are clearly noteworthy in terms of vaccine hesitancy. Feelings of regret after getting the COVID‐19 vaccine may leave society vulnerable to many vaccine‐preventable infectious diseases in the future. In fact, looking at this event through the lens of behavioural sciences can provide more powerful insights. Behavioural sciences reveal that individuals are often inclined to make decisions based on their feelings and intuitions rather than existing scientific information [35]. Similarly, Toomey (2023) demonstrates, through social change models, that in a new decision‐making process, scientific and new information often loses ground to beliefs and feelings. Research shows that feelings of regret stemming from previous experiences have an invisible power over new decisions [36]. In light of this information, it can be said that feelings of regret have the potential to silently implant a negative image of vaccines into individuals' subconscious. This situation may make it more difficult to protect public health and control vaccine‐preventable infectious diseases in the coming years. It can be said that health policies need to be developed to reduce this situation.

When the variables related to participants' levels of trust/distrust towards the health system were examined, it was found that participants who had recovered from COVID‐19 had higher levels of trust in the health system than others. Those who have recovered from the illness may have gained a better understanding of the importance of the healthcare system and treatments in the face of the severity of the disease. In fact, it can be said that this situation is consistent with the conceptual structure of the health belief model [37]. The increased perceived severity of the disease among those who experienced it may have supported their trust in the health system. In contrast, those who have never experienced the disease have low trust in the health system [32].

Among the participants, those who experienced adverse effects from the COVID‐19 vaccine were found to have high levels of distrust in the healthcare system. Similarly, Souvatzi et al. (2024) reported that negative outcomes of medical treatment reduce trust in the healthcare system [38]. To mitigate this, providing individuals with detailed and honest information about the possible adverse effects of treatments and vaccines could positively impact their level of trust in the healthcare system.

When the participants' future intentions to get vaccinated and their levels of trust in the health system were examined in the study, those who stated that they would get a recommended vaccine for any future pandemic had higher levels of trust in the health system. This finding remarkably demonstrates the relationship between trust or distrust in the health system and health behaviours. Similarly, Antinyan et al. (2021) reported that the perception of trust/distrust in the health system strongly influenced individuals' intentions to accept vaccination or treatment [39]. Similarly, a study conducted in Romania also found that trust in the healthcare system decreased after the pandemic, negatively affecting the acceptance of certain public health interventions [40].

In the study, there was a weak, positive correlation between participants' regret about the COVID‐19 vaccine and their level of distrust in the health system. As the level of regret increased, the level of distrust also increased. Partially similarly, previous studies have found a correlation between regret felt after various treatments and individuals' levels of trust in the health system [41]. Similarly, in research conducted on vaccine hesitancy, it is known that the perception of trust/distrust in the health system affects attitudes towards vaccines [42, 43]. When the quantitative data obtained at this point are evaluated together with the qualitative data, it can be said that the level of distrust in the healthcare system is influenced by two different sources of regret regarding the COVID‐19 vaccine, as seen in the data integration table. Specifically, the anxiety and unease created in individuals by the unknown future effects of the COVID‐19 vaccine (the first theme) are largely reflected as distrust in the healthcare system. The second theme is less related to the level of distrust in the healthcare system. While the first theme emphasises risk perception, the second theme is based on feelings of disappointment and unmet expectations. Interestingly, some studies conducted in Türkiye before the pandemic reported that vaccine acceptance was generally positive and trust in the healthcare system was at a moderate level [24, 44]. In this context, the current findings may be a reflection of the changes in perceived trust in the healthcare system and attitudes towards vaccines that emerged during the pandemic, as also indicated in the literature [6, 45].

### 4.1 Limitations

This study has several limitations. Firstly, the sample was taken from Western Türkiye, a region with a relatively higher level of sociocultural development compared to some other parts of the country. Although healthcare services are provided equally across the country through a centralised health system, regional differences in health awareness and access to health information can affect attitudes toward vaccination programmes and trust in the health system. Therefore, caution should be exercised when generalising the findings to the broader Turkish population. Second, the data were collected using self‐report questionnaires, which may have introduced social desirability bias. Finally, the reasons for COVID‐19 vaccine regret were limited to participants' responses to the open‐ended questions included in the questionnaire. A mixed‐methods approach could have provided a more comprehensive understanding of the underlying reasons for COVID‐19 vaccine regret.

## 5 Conclusion

This study reveals a high incidence of COVID‐19 vaccine regret and distrust of the healthcare system among participants. Qualitative findings revealed that the main reasons for COVID‐19 vaccine regret were ‘concern about long‐term effects’ and ‘perception that the vaccine is unnecessary.’ The findings suggest that COVID‐19 vaccine regret and distrust of the healthcare system have the potential to influence future vaccination intentions. Additionally, factors such as the death of a relative from COVID‐19, COVID‐19 vaccine preference, and adverse effects of the vaccine were found to be associated with the level of vaccine regret. Furthermore, COVID‐19 disease experience and adverse effects of the vaccine were related to the level of trust in the healthcare system. Based on these findings, it can be said that the phenomenon of COVID‐19 vaccine regret will make it more difficult to combat future pandemics and infectious diseases. This phenomenon appears to be a significant factor influencing vaccine hesitancy. Strategies to reduce COVID‐19 vaccine regret and distrust in the healthcare system urgently need to be developed. In addition, it can be said that the image of healthcare services needs improvement.

## Author Contributions

**Ali Tayhan:** supervision, formal analysis, investigation, conceptualisation, writing – review and editing, resources, roles/writing – original draft, project administration, data curation, software. **Bilge Azaklı:** investigation, formal analysis, data curation, validation, resources, visualisation, roles/writing – original draft, methodology.

## Funding

The authors have nothing to report.

## Ethics Statement

Ethical approval was received from the Health Sciences Ethics Committee of Manisa Celal Bayar University to conduct this research (numbered 2187 and date 27/12/2023). Potential participants were informed about the study, and then verbal and written consent was obtained from those who volunteered. Participants were told that they could withdraw from the study at any time. Detailed information was provided through a voluntary consent form. The principles of the Declaration of Helsinki, ethical rules and human rights were meticulously adhered to at every stage of the research.

## Conflicts of Interest

The authors declare no conflicts of interest.

## Data Availability

The data that support the findings of this study are available from the corresponding author upon reasonable request.
